# Understanding determinants of infection control practices in surgery: the role of shared ownership and team hierarchy

**DOI:** 10.1186/s13756-019-0565-8

**Published:** 2019-07-15

**Authors:** Rachael Troughton, Victor Mariano, Anne Campbell, Shehan Hettiaratchy, Alison Holmes, Gabriel Birgand

**Affiliations:** 1 National Institute for Health Research, Health Protection Research Unit in Healthcare Associated Infection and Antimicrobial Resistance at Imperial College London, Hammersmith Campus, 7th Floor Commonwealth Building, Du Cane Road, London, W12 0NN UK; 2 Major Trauma Centre, St. Mary’s Hospital, Imperial College Healthcare NHS Trust, Praed Street, London, W2 1NY UK

**Keywords:** Surgical site infection, Infection control, Healthcare settings, Behaviours, Social determinants, Qualitative

## Abstract

**Background:**

Despite a large literature on surgical site infection (SSI), the determinants of prevention behaviours in surgery remain poorly studied. Understanding key social and contextual components of surgical staff behaviour may help to design and implement infection control (IC) improvement interventions in surgery.

**Methods:**

Qualitative semi-structured interviews were conducted with surgeons (*n* = 8), nurses (*n* = 5) theatre personnel (*n* = 3), and other healthcare professionals involved in surgery (*n* = 4) in a 1500-bed acute care London hospital group. Participants were approached through established mailing lists and snowball sampling. Interviews were recorded and transcribed verbatim. Transcripts were coded and analysed thematically using a constant comparative approach.

**Results:**

IC behaviour of surgical staff was governed by factors at individual, team, and wider hospital level. IC practices were linked to the perceived risk of harm caused by an SSI more than the development of an SSI alone. Many operating room participants saw SSI prevention as a team responsibility. The sense of ownership over SSI occurence was closely tied to how preventable staff perceived infections to be, with differences observed between clean and contaminated surgery. However, senior surgeons claimed personal accountability for rates despite feeling SSIs are often not preventable. Hierarchy impacted on behaviour in different ways depending on whether it was within or between professional categories. One particular knowledge gap highlighted was the lack of awareness regarding criteria for SSI diagnosis.

**Conclusions:**

To influence IC behaviours in surgery, interventions need to consider the social team structure and shared ownership of the clinical outcome in order to increase the awareness in specialties where SSIs are not seen as serious complications.

**Electronic supplementary material:**

The online version of this article (10.1186/s13756-019-0565-8) contains supplementary material, which is available to authorized users.

## Introduction

Surgical site infection (SSI) is one of the most common healthcare associated infections (HAIs), accounting for approximately 20% of all HAIs among hospitalised patients in Europe [1]. Patients with an SSI have a 2–11 times higher risk of death, compared with operative patients without an SSI [2, 3], yet SSIs are among the most preventable HAIs [4].

Local context and culture, characteristics of teams, and local systems, all impact on frontline practice in the OR [5], and challenging existing norms and practices may be difficult [6]. Risk factors for post-operative infections are linked to patient characteristics, the surgical procedure, the operating room (OR) environment and post-operative care. Many national and international organisations have produced evidence-based recommendations to mitigate SSI risk, which are regularly reviewed and updated [6–9]. However, these guidelines have tended to focus on healthcare professional knowledge, skills, and motivation, with neglect of the wider social and organisational context that shape practices. Consideration of the multiple factors that impact on practice, and integration of strategies across the diverse categories of professionals implicated in preventing SSIs, are likely to be critical in designing effective interventions to optimise surgical care [10].

When designing and implementing quality improvement interventions, involving all stakeholders (multidisciplinary/multi-professional) early in the process, and considering relationships between individuals has been shown to increase chances of success [11–14]. Integrating this approach in interventions to improve IC practices in surgery requires research into the social and contextual determinants of surgical staff behaviour. An understanding of how to design interventions that address the barriers to implementing IC practices, and which are likely to be implemented and adopted by healthcare professionals have also to be considered [14, 15].

The study reported here explored the social determinants of SSI prevention in the hospital setting and adds to the existing knowledge in the field. To date, previous studies into SSI prevention have largely used quantitative methods to assess the impact of or compliance with strategies. Few studies have explored the factors that influence surgical staff behaviours and IC practices [16, 17]. Qualitative research is now needed to explore the drivers of compliant and non-compliant IC behaviour in surgery and the impact of social and organisational context on practices.

The present study, that focused on surgical staff, aimed to explore: (i) attitudes and perspectives of healthcare professionals towards perioperative infection risk/prevention, and shared ownership of quality indicators/surgical outcomes, (ii) barriers to and facilitators of adherence to quality improvement interventions in SSI prevention, (iii) determinants of IC behaviours including contextual, environmental, and social factors.

## Methods

### Setting and study design

This study was conducted in three hospitals belonging to the Imperial College Healthcare National Health Service Trust (ICHNT) hospital group across West London, United Kingdom. ICHNT is a multisite, 1500-bed healthcare delivery organization that operates in partnership with Imperial College London. All hospitals within ICHNT operate within one organisational structure. SSI prevention and surveillance activities are coordinated through a multidisciplinary team including surgeons, nurses, antimicrobial pharmacists, IC teams, and infectious disease and medical microbiology teams. ICHNT currently undertakes surveillance on SSIs in total hip and total knee arthroplasty, and cardiac surgery. An audit on SSI rates part of a national programme “Getting It Right First Time” (GIRFT), was launched during the study. Getting It Right First Time was designed to improve the quality of care within the NHS by reducing unwarranted variations [18].

We designed a qualitative study using semi-structured interviews with a range of stakeholders in order to describe workplace culture around SSI prevention behaviours. The topic guide for the semi-structured interviews was developed by the authors of this study in collaboration with experts involved in the intra and perioperative SSI prevention (Table 1).

**Table 1:** Interview guide, including supplementary questions

Role of infection control in preventing SSIs	• In your view, what are the most important factors in preventing SSI? • What activities in your day-to-day work do you do to help to prevent SSI?
Knowledge of SSI preventive measures	• Are you aware of any policies or guidelines that apply to SSI prevention? • How significant a problem do you feel SSIs are in this Trust?
Diagnosis and surveillance	• How are SSIs usually diagnosed and communicated among staff at an individual patient level and globally? • How would you and others know if there was a problem with SSIs here? • What happens to patients after discharge? How would you know if they were infected?
Barriers to compliance SSI preventive measures	• How easy is it for you to prevent SSI? Is it ever difficult? Why? • Thinking specifically about policies or guidelines that apply to SSI prevention, to what extent do you feel these are practicable?
Facilitators to compliance SSI preventive measures	• Who is accountable for SSI prevention, in your opinion? • What sort of feedback do you get on these activities - at an individual or team level? ◦ How often does this occur? Is the feedback confidential or open? ◦ How often do you get the chance to watch others work? • What do you see as important in terms of you colleagues’ roles in preventing SSIs? ◦ Does this work well or are there any problems? Why? ◦ Do you feel you are in position to question the infection control behaviours of your colleagues and superiors? ◦ How would you describe the culture of transparency around SSI here? • Is there anything you feel can be done to improve SSI rates in your hospital? • Is there any new technology, gadgets, apps etc. that you know of that relate to SSIs?

*Abreviation*: *SSI* Surgical site infection

### Inclusion criteria and recruitment

To be eligible for the study, participants had to be either surgical professionals (defined as surgeons, anaesthetists, intensivists, microbiologists, pharmacists, nurses) with regular contact with patients and/or participating in the care of surgical patients inside or outside the OR, or involved with the surveillance and prevention of SSIs (surveillance nurse or members of the IC team).

### Qualitative semi-structured interview methodology

Between May 2017 and July 2018, 68 healthcare professionals were invited to participate in the study. Participants were approached through established distribution lists, and then identified purposively through snowball sampling to ensure representation from a range of stakeholders. Participants were contacted by email and invited to contact the researcher if they wanted to participate in the study. Reminders were sent after 2 weeks. All participants who responded to this invitation were invited to a face-to-face or telephone interview at a time convenient to them. Participants were provided with an information sheet detailing the study aims and methods and a consent form at least 24 h prior to interview. Interviews were conducted by RT, a researcher, and VM, a former theatre nurse. Face-to-face interviews were conducted on hospital sites. Interviews were carried out outside of work time unless the participant had chosen to make arrangements with their manager. Recruitment was allowed to continue until thematic saturation was achieved, i.e. until no new major themes were identified in the data [19]. Data analysis proceeded simultaneously with data collection. All interviews were audio recorded and transcribed verbatim by a third party commercial company under a confidentiality agreement. The audio files were deleted once transcribed copies had been checked for errors.

### Analysis

The transcribed data were uploaded into NVivo®, QSR International Ltd., Version 11. Data were analysed using a thematic analysis approach [20], drawing on the constant comparative method [21]. A selection of transcripts was first open coded inductively, with codes created from the patterns and themes emerging from the data, and an initial coding frame developed. This coding frame was then applied to subsequent transcripts and iteratively refined as new codes were identified. The authors (R.T., G.B.) discussed the content of the categories until no inconsistencies existed and a shared understanding was reached in order to reduce researcher bias and strengthen the internal validity. In order to understand any possible biases in the data collection and analysis stage, researchers (R.T., V.M. and G.B.) kept reflective journals which documented their thought processes at each stage.

### Ethical considerations

Approval from a Health Research Authority was obtained for this study (16/HRA/5160 for IRAS project ID 193411). Both participation in the study and the data collected were treated as confidential. Any identifiable data were redacted from the transcripts and participants were referred to by a generic name detailing only the staff group and specialty e.g. cardiac surgeon 1.

## Results

**Fig. 1:**
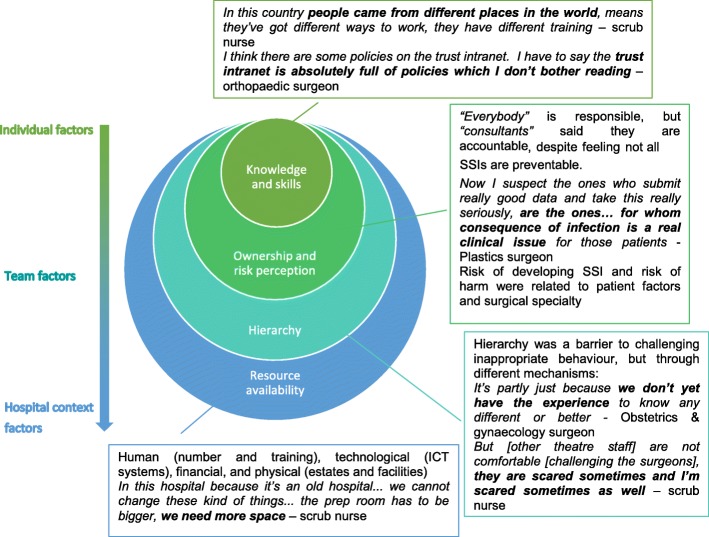
Drivers of SSI prevention behaviour at individual level, team level, and the wider hospital context

Of the 68 health care professionals invited to take part to the study, 22 (32%) responded to the invitation. Among these, interviews were arranged with 20 participants. The staff interviewed represented a wide range of specialties and expertise (Additional file 1: Table S1). All participants were interviewed face-to-face for an average duration of 46 min each (range: 10 min - 2 h 14 min). The analysis identified four key themes in relation to SSI prevention derived from data collected: (1) knowledge, skills, and self-efficacy, (2) ownership and risk perception, (3) the culture of hierarchy and apprenticeship, and (4) resource availability. These drivers covered a spectrum of levels from individual factors (knowledge, skills, risk perception, and ownership) through team factors (hierarchy) up to external context factors (human, financial, physical, and technological resource availability) (Fig. 1, Additional file 1: Table S2).

### Lack of self-efficacy regarding SSI prevention and diagnosis

Forming the bedrock of staff attitudes to SSI is their existing knowledge and skills around SSI prevention and diagnosis. While most staff reported that they felt well equipped to prevent SSIs, some reported being asked to perform tasks they had not been trained for at all, had not been recently trained for, or were not confident in the training they had received (Table 2, Q1). Some relied on their original training, or from previous workplaces:

*“ … when I scrub … , I mean with the aseptic technique … as I’ve learned in university … In this country people came from different places in the world, means they’ve got different ways to work, they have different training. … but you still can do the same work as I used to do in a different way, because we have been trained in another country”.* Scrub nurse

**Table 2:** Themes and illustrative data

Theme	Sub-theme	Illustrative quotation
Knowledge and skills	Lack of continuous training	Q 1: *So, if I do a wound care dressing as what I knew from university … all like the basics that I’ve learned from taking the degree … I don’t think this is standardised across nurses … I cannot remember that someone has shown me that this is how you should do wound care. -* Critical care nurse
	Lack of peer sharing	Q 2: *When I do the dressing no one is there, so anyone who’s checking the dressing in that closed curtains, actually in my head I don’t know how they’re doing it. Do they do it the way I do, is it my practice it is the best, I don’t also know. -* Critical care nurse
	Policies, guidelines, or high-quality evidence to refer to	Q 3: *Before that I can only suggest maybe we shouldn’t be doing this, other people can see you like, oh, smartass is coming and saying oh, we should do this or that and what is your evidence? Well, there are multiple evidence, WHO guidance from 2016, for example... -* Vascular surgical registrar
Ownership	Surgeon’s reputation	Q 4: *if you have lots of wound infections, that would look bad on you, because it suggests that there may be a problem with your technique. -* Cardiac registrar
	Clinical team responsibility	Q 5: *Everybody who the patients, along the patient’s pathway is responsible for ensuring and in, especially the patient of course themselves, but everybody that the patient comes into contact with. -* Theatre personnel
	Feedback	Q 6: *“this happens in a cyclical manner in every hospital I’ve ever worked in, but there will always be a period of time where there’s lots of wound infections … And everyone will come up with a series of steps to try and reduce that, and what will happen is, the infection will go away and everyone will say it’s because of all we’ve put in place … And actually I think it’s because everyone’s more aware of what’s going on, and when you’re more aware of what’s going on, every step is better …*” *-* Cardiac registrar
	Patient related factors	Q 7: *The majority of the SSIs we see are those who have, like, perforate, so they have a hole in the bowel, and then faeces, and then you do a big cut, a laparotomy, and then when you close that off there’s a high rate of an SSI because it’s a contaminated wound. But that you can’t control for. -* General surgery registrar
	Conflicting priorities	Q 8: *there are times where, for example, the patient arrests in theatre which has happened, and we have opened a chest without any preparation whatsoever, you just, normal gloves which are not sterile, but that’s a different situation where the patient’s essentially dead, or, but otherwise we have to go through the steps. -* Cardiac registrar
	Procedure types	Q 9: *in … elective surgery you know you should have essentially a close to zero infection rate in the majority of patients, unless they have got some significant problem. For trauma surgery I think that’s very different because you’re starting with a, at least a contaminated wound … -* Plastics consultant
	Consequences for patients	Q 10: *Now I suspect the ones who submit really good data and take this really seriously, are the ones who, for whom consequence of infection is a real clinical issue for those patients compared to, so that’s why in cardiac and orthopaedics certainly, for them, infection’s an absolute disaster. Whereas if you’re a general surgical patient, you get a of bit wound infection, that’s normally not a big deal because you can clear it up. -* Plastics consultant
	The power of awareness	Q 11: *… this happens in a cyclical manner in every hospital I’ve ever worked in, but there will always be a period of time where there’s lots of wound infections … And everyone will come up with a series of steps to try and reduce that, and what will happen is, the infection will go away and everyone will say it’s because of all we’ve put in place … And actually I think it’s because everyone’s more aware of what’s going on, and when you’re more aware of what’s going on, every step is better … -* Cardiac registrar
Culture of hierarchy	Fear of offending or provoking a negative reaction	Q 12: *Oh, definitely. There is hierarchy … there are people who … will do their way anyway, without, whatever you tell them it doesn’t, it wouldn’t really matter. [It’s harder to challenge someone] who’s very senior, yes. Yeah, it’s harder to challenge... -* Vascular surgical registrar
	Challenge with tact and a non-judgemental way	Q 13: *they’re not upset when they do it, because they, you have to do it with tact. You can’t be rude, aggressive or loud or do things with an attitude … your body language, your tones, your attitude be of one that they would listen to … you kind of whisper little things in the ear and you make a little joke about it and you be, throw in a bit of sarcasm on the sly and things like that … it’s a personality thing … -* Theatre coordinator
	Staff member’s official remit or area of expertise	Q 14: *It’s not so much that whoever holds the knife, as you know, you have met [nurse] … [nurse] never holds the knife but holds a lot of power...is empowered to actually cause a fuss if people are doing the wrong thing … -* Cardiac surgical registrar
		Q 15: *It depends if it’s my specialty or not. If it’s for example a simple mistake of wrong prophylaxis I will challenge that … So, I think the hierarchy is only valid if you are within the team, if you’re outside the team and if you are a consultant in another specialty, you are confident in what you are doing without obviously being arrogant. -* Microbiology consultant
		Q 16: *Personally, I feel comfortable, but I think that’s probably because I work within that specialty. If I was a junior pharmacist on the ward, who wasn’t specialising in infection, I think I would find it difficult to challenge perhaps a consultant who was coming along on a ward round with a watch or a suit jacket on, but to me, it doesn’t really faze me to do it. I’m used to it. -* Infection pharmacist
Resources – human, financial, physical	Skill mix	*Q 17: In cardiac surgery the SSI rate, it goes up and down … I think it’s a just a problem with the team being really incredibly busy, and not having enough work force to cope with the amount of [surveillance] work. -* Microbiology consultant
		Q 18: *There was an interview and they found a person who did tissue viability nurse’s courses and other, so she will be probably over qualified to do only dressings … -* Vascular surgery
	Availability of other specialist staff as a resource	Q 19: *Every hospital has a different policy and the policy is based on what the microbiologist feels is the current, most important bacterial infections but also we have meetings between different departments to discuss that. The microbiologist tends to give the majority of the advice with some input from clinicians, so it’s mainly the microbiologist who decides what the antibiotic prophylaxis is. -* Cardiac registrar
		Q 20: *I think it is well organised team for surgical infection site in our trust starting from the nurses and to surgeon between, for example in our department of orthopaedic as well as the plastic surgeon and the microbiology department and as well as pharmacy. -* Orthopaedic surgeon
		Q 21: *… the referrals that I see, I don’t get referred that many but that doesn’t mean to say that it’s not, they’re not occurring or not being reported but I know there has been a slight increase in the number of SSIs occurring recently … because the GI surgery, they refer quite a lot and they want 7our opinion quite often, whereas other types of surgery … never refer. -* Tissue viability nurse

Overall, staff did not tend to get feedback on the majority of their practices, and very rarely at an individual level (Table 2, Q2). This led to feelings of confusion, lack of self-efficacy and reluctance to carry out tasks:

*“ … I notice also if the nurse doesn't want to do the dressing they will be very reluctant to call you when the dressing is down, and they won't do it, they won't take it down.”* Vascular registrar

One particular knowledge gap highlighted was that some surgeons were unaware, until the “Getting It Right First Time” (GIRFT) national audit of SSI rates, of the criteria for diagnosing an SSI. Participants who had undergone specific training in the diagnosis criteria for SSIs felt that most other staff were not familiar with the definition, and therefore not recording SSIs correctly in the notes.

*“It’s the education of that person and how they can define or diagnose it as a wound infection has also a part to play … since doing the audit … I’ve learnt how to define it and the parameters … We could definitely do with more education around SSIs, what SSIs are … ”* GIRFT surgeon registrar 2

When asked which policies and guidelines they knew for SSI prevention, many staff cited guidelines produced by the National Institute for Health and Care Excellence (NICE) and World Health Organisation (WHO). In addition to an official remit or expertise, staff found having policies, guidelines, or high-quality evidence to refer to was helpful in supporting their position when discussing SSI prevention [22] (Table 2, Q3). However, they reported that the process of obtaining these policies was either unclear or too inconvenient to be of use in regular practice:

*“I have to say the trust intranet is absolutely full of policies which I don’t bother reading. I will do what we’re told to do if it is discussed in our audit meeting or, for example by the ward matron or whoever is the leader on it.”* Orthopaedic consultant

### Ownership of SSIs

Most staff stated that the whole clinical team was responsible for SSI prevention (Table 2, Q7), and some extended this to the cleaning staff and patients themselves. However, several surgeons explicitly stated that accountability for SSI rates rests with the consultant surgeon. This was echoed by the surgeons’ assertions that high SSI rates would negatively impact their professional reputation among peers (Table 2, Q4). Surgeons reported simultaneously feeling both solely accountable but not solely responsible for infection rates. The leadership of the whole team was assigned to the matron, accountable to support and supervise team members:

*“So I don't expect every nurse will know about wound … I expect from the matron of the ward just to support them and supervise if there is a need … ”* Vascular registrar

The sense of ownership over SSI occurence was closely tied to how preventable staff perceived infections to be, and to the overall clinical outcomes for patients. Participants reported that some SSIs are not preventable, especially in trauma or dirty surgery. Some examples included when a patient has co-morbidities such as frailty or uncontrolled diabetes, or has complications such as bowel perforation (Table 2, Q7). The risk of wound contamination and subsequent SSI following general surgery was perceived high and hard to avoid, leading to less intensive SSI prevention efforts (Table 2, Q9). In contrast, practices in orthopaedic surgery were perceived as being extremely rigorous and strictly enforced:

*“ … if I am inside the theatre and I have a staff which is from general surgery you can see their attitude or their behaviour towards dealing with the operation is totally different. So they don’t take, for example for us we take everything seriously … ”* Orthopaedic registrar

Participants explained the strict approach in orthopaedic surgery by the burden of SSIs in prosthetic joint infections (Table 2, Q10).

Participants discussed how they perceived data to be vital for quality improvement, as it can highlight problem areas and allow the causes of high rates to be investigated. When surveillance data are unavailable, participants reported how they relied on their own ad-hoc experience treating patients with SSIs, and complications data discussed in morbidity and mortality meetings. However, healthcare professionals not involved in the post-operative follow-up (e.g. non-surgeon OR staff) were unaware of individual patient outcomes.

The later interviews revealed that some participants were informed of SSI rates by the results from the recent “Getting It Right First Time” (GIRFT) national audit. One participant reported that little or no attention is paid to suspected high SSI rates unless solid evidence of a problem is highligted:

*“ … suddenly the problem is made visible because it was before but no one notices or people pretended not to notice this, it will make them to change the practice … ”* Vascular registrar

When data on outcomes is available there is not only improved buy-in from staff for the formal action plan, but staff in general become more sensitized to the risk of SSI and as a result every aspect of care is improved (Table 2, Q6).

### The culture of hierarchy and apprenticeship

Participants working within the OR or within surgical specialties reported difficulty in reminding best practices tochallenging staff they perceive to be superior to them, usually consultant surgeons. The reasons behind the reluctance to challenge surgeons varied based on professional groups. Among surgeons, the training is perceived to be an “apprenticeship”, in which the consultant is the master and the registrar the student. The reason for a lack of nudge from junior to senior surgeon is partly based in the assumption of superior knowledge and experience of the consultant:

*“ … I think as a trainee, particularly when you’re, you’ve got a weak evidence base behind you, it’s very hard to challenge, and I think it’s very hard to challenge even as a consultant I think it’s hard to challenge peer behaviour … when you’re a trainee, the factor that stops you challenging or questioning is hierarchical normally … they’re a boss, he or she knows more than I do because I can’t tell them from the evidence that what they’re doing is wrong or right … ”* Plastics consultant

This differs from other staff groups working within the operating theatre or surgical specialty, for whom the main barrier was a fear of offending, or even provoking a negative reaction from the surgeon:

*“ … sometimes I just prefer not to say anything, I know it’s not right, but I just prefer to not say anything … I will get nervous, because … we say that they have the knife, they have the power … ”* Scrub nurse

This is also a secondary barrier for junior surgeons, or even consultant surgeons challenging each other. The availability and accessibility of high-quality evidence (SSI prevention policy and guidelines) to refer to was felt helpful when discussing inappropriate practices or behaviours with superiors. These supports embolded stakeholders to act and helped to make the discussion less individual and more general and academic.

Confidence in reminding others on best practices for almost all participants was rooted in their perceived ability to express themselves in a way that seems informal, unconfrontational, and friendly (Table 2, Q13). This partly depended on their existing relationship to the person, with some describing a “good relationship” as a facilitator for successfully challenging the person. The addition of humour was also highly valued. Many participants disputed the word “challenge”, instead referring to discussions or other less confrontational exchanges.

### Resources – human, financial, physical

Participants reported that staffing levels and financial investment were well-recognised factors that influence IC behaviour. More nuanced factors were also reported, including skill mix, ward layout, and unsuitable facilities. One important facilitator in preventing, diagnosing and treating SSIs was the availability of transversal specialist staff as a resource for example connections with microbiologists, pharmacists, tissue viability and IC. However, this multidisciplinary work varied across surgical speclialities, with some referring patients to tissue viability more often than others (Table 2, Q21).

As well as staffing issues, switches in information systems disrupt workflow and can create new problems without any tangible benefits to end users, at least in the initial stages.

Physical infrastructure also had an impact on behaviour. Overcrowding in the wards, poor state of repair and issues with storage affected IC practices in surgery.

In terms of resources required to make surveillance more sustainable, participants suggested to create a specific role, adding the duty to somebody’s job description, providing more funding, and utilising electronic records for easier data capture.

## Discussion

More than individual skills and training, contextual, environmental and social aspects seem to represent key determinants of behaviours for SSI prevention. IC behaviours are determined by the ownership of SSI, the perception of risk of patient harm, and hierarchy relationships (Table 3). Even when staff intend to comply with best IC practices they are sometimes constrained by the infrastructure and resources available.

**Table 3:** Rules of infection control practices in surgery

Rules	Descriptions
Awareness through the ownership of SSI	• Accountability for SSI, vision on consequences to patients, and individual impact on reputation hold by the senior surgeon.
	• Surgeons awareness relying on their own ad-hoc experience treating patients with SSIs, and complications data discussed in morbidity and mortality meetings.
	• For non-surgeon staff, awareness through data on post-operative outcomes if available improving buy-in for formal action plan.
Perceived SSI preventability driving behaviours	• SSI more tolerated in high risk surgery or patients and put in the context of fatalism. This leads to specialty or patient driven behaviors and more flexibility in infection control practices.
Hierarchy leading tolerance of poor IC practices	• Assumption of superior knowledge and experience by the senior surgeon, and fear of offending, or even provoking a negative reaction from the surgeon lead to a culture where poor IC practices are tolerated.

*Abreviations*: *SSI* Surgical site infection, *IC* Infection control

A lack of self-efficacy was found to be a barrier for the implementation of best practices. Knowledge renewal, audit and feedback were described as the main reasons for lack of confidence in routine cares performed. Staff often rely on practices they acquired during their initial education and training. However, the field of surgery is in constant evolution. Despite efforts for continuous training of clinical staff, some categories slip through the net. Surgery requires highly qualified resources. As described in interviews, hospitals and surgical departments currently have insufficient resources to cope with increased demand. Thus, we may hypothesise patient care and ward activities are prioritised by the organisation management over continuous training. Education and training involving frontline surgical staff should be programmed at the organisation level, including team and task orientations and knowledge/ competency assessments [23].

One specific knowledge gap reported by some surgeons was a lack of awareness of the criteria for diagnosing an SSI, before the occurrence of the “GIRFT national audit”. Participants who undergone specific training in the diagnosis criteria for SSIs felt that most other staff were not aware of the definition, and therefore not recording SSIs correctly in the notes. Improving staff skills in SSI diagnosis would lead to more accurate recording, more standardised surveillance data, and ultimately better patient care. Low inter-rater reliability of SSI diagnosis is a recognised problem [24], that might be improved through training. The involvement of surgical staff in SSI surveillance programs and the regular cross-hospital evaluations of diagnostic accuracy through case-vignettes, as conducted in Germany in the KISS surveillance network, probably would improve the knowledge around the physiopathology of SSI and their diagnostic criteria [25].

Ownership over the development of SSIs was an important determinant in IC behaviour, directly associated with the perceived preventability of infections. When staff perceive an SSI to be preventable, they feel an increased ownership regarding the outcome. Participants’ perceptions of the risk were based not just whether or not the patient develops an SSI but how much harm that SSI might cause. SSI rates appeared to be considered as more of a process than an outcome measure by surgical teams, the outcome measure being ultimately the overall harm done to the patient.

Participants perceived the risk of developing an SSI in gastro-intestinal surgery as high. However, the harm caused to patients by these infections was felt relatively low or easily manageable, in contrast with orthopaedic surgery. A recent study estimated SSIs in large bowel surgery to contribute 38% of SSIs in England [26]. Previous studies have shown reductions of SSI rates in large bowel surgery of up to 50% after introduction of a prevention bundle [27], challenging the view of some participants that these infections are generally not preventable. Including morbidity and mortality related to SSIs in surveillance data would facilitate the interpretation of consequences by clinicians.

Despite being bypassed from patients outcomes and SSI rates, OR staff felt SSI surveillance important in achieving quality improvement and general buy-in from staff. However, indications of who should be accountable for this data was less clear. Participants who were surgeons reported feeling solely accountable but simultaneously not solely responsible for infection rates, highlighting a degree of cognitive dissonance among this participant group. This tension may explain, in part, the difficulties in encouraging transparency around SSI diagnosis and surveillance as surgeons cannot reconcile the accountability for high rates, which they accept to be theirs, with their belief that SSI prevention is neither completely their responsibility, nor always completely possible. An appropriate sharing of surveillance data may help foster a sense of team accountability by reflecting the complex responsibility in SSI prevention [16]. The strategy of output dissemination should meet the stakeholder needs, be timely, high quality, with accessible content and delivering interpretation and recommendations. Many individuals or collective communication support may be used including bulletins, reports and interactive web-based tools. However, the engagement of key staff may come from face-to-face conversations providing opportunities to learn from results.

Surgeons in this study spoke of their training as an apprenticeship in which they learn from the greater experience of the consultants and from their own experiences. Power relationships are prominent in surgery and can lead to fear and silence, ultimately allowing unsafe practices to go unchallenged [28]. Previous studies have highlighted the use of “quiet words”, humour, and bantering to help diffuse tensions when challenging inappropriate behaviour [29] which were also common in this study, however brutal reprimands and public humiliation were only mentioned as rare and undesirable consequences of challenging seniors. IC staff sometimes decided not to challenge inappropriate behaviours in favour of maintaining more harmonious relationships in the long term [29], whereas the IC, microbiology and pharmacy staff in this study reported that they would have no hesitation in challenging others.

The issue of hierarchy among healthcare professionals has been widely described [28, 30]. In addition to previous studies on surgical staff behaviour, the willingness of surgeons to question seniors was related to doubts about their own understanding, ignoring the possibility that practices and knowledge may have evolved over time and across settings. A published review noted a particularly low regard for the knowledge of nurses [28], which was evident to some extent in the experiences of the scrub nurse in this study. Surgeons, experienced and stable professionals are positioned in a dominant situation. In order to access the combined knowledge of the team around them, consultants need to encourage staff to speak up if they observe deviating practices. By referring to policies, literature, guidelines, and data on infection rates, staff can “collect facts” to support their point of view [6]. Staff could also circumvent difficult hierarchies by “selecting the person” appropriately, for example by asking somebody who does feel comfortable challenging the individual to discuss the issues raised.

The availability of human and financial resources and the existing infrastructure in terms of IT systems, built environment were clearly explained as being key determinants of IC behaviours by a majority of participants. All of these additional factors mean that even if staff have the knowledge, skills, ownership, self-efficacy, and team support in place to enable best practice, there are still circumstances outside their control which can help or hinder these efforts. Moreover, on top of any direct impact of these factors on IC practices, lack of investment in estates, facilities, and infrastructure can also be construed as a lack of interest in the needs and requirements of staff [31].

The main limitation of this study was that it was conducted in three teaching hospitals, with reliance on self-reporting. As the study setting was a large hospital group in London, there may be some factors specific to this setting that may influence the generalisability of the findings.

## Conclusion

This study enlarges the scope of research on SSI prevention and extends our understanding of how to optimise perioperative IC behaviours by describing contextual determinants. The self-efficacy, ownership of SSI and the social team structure must be recognised and addressed in the SSI prevention agenda, and be part of future implementation staregies in surgery.

## Additional file

Additional file 1: **Table S1.** List of study participants. **Table S2.** Sample coding framework – Knowledge and skills theme. (DOC 81 kb)

## Data Availability

The datasets generated and analysed during the current study are not publicly available due to confidentiality clauses but anonymised versions are available from the corresponding author on reasonable request.

## Abbreviations

HAIs: Healthcare associated infections
IC: Infection control
IT: Information technology
KISS: Krankenhaus infektions surveillance system
OR: Operating room
SSI: Surgical site infection
