# Supplementary material for: Understanding determinants of infection control practices in surgery: the role of shared ownership and team hierarchy
Source: Antimicrob Resist Infect Control. 2019 Jul 15;8:116. doi: 10.1186/s13756-019-0565-8 (PMC6631607; doi:10.1186/s13756-019-0565-8)
Supplement: Supplementary file 1 — Table S1. List of study participants. Table S2. Sample coding framework – Knowledge and skills theme. (DOC 81 kb) [file 13756_2019_565_MOESM1_ESM.doc]

**Supplementary Material**

**Understanding determinants of infection control practices in surgery: the role of shared ownership and team hierarchy.**

Corresponding Author:

Gabriel BIRGAND

7th Floor Commonwealth Building

Imperial College London

Du Cane Road

London, W12 0NN

Tel: 020 3313 32732

Email: [g.birgand@imperial.ac.uk](mailto:g.birgand@imperial.ac.uk)

**Table S1 List of study participants**

| **Specialty** | **Staff group** | **Experience** | **Interviewed by** | **Gender** |
| --- | --- | --- | --- | --- |
| Anaesthetics | Anaesthetist | 13 | RT | M |
| Microbiology | Microbiologist | 5 | RT | M |
| Tissue viability | Nurse | 10 | RT | F |
| Orthopaedic | Nurse | 10 | RT | F |
| Critical care | Nurse | 16 | VM | F |
| General surgery | Nurse | 4 | VM | M |
| A&E | Nurse | 3 | VM | M |
| Pharmacy | Pharmacist | 8.5 | RT | F |
| General surgery | Surgeon | 3 | RT | M |
| Vascular | Surgeon | 10 | RT | F |
| Cardiac | Surgeon | 10 | RT | M |
| Orthopaedic | Surgeon | 15 | VM | M |
| Neurology | Surgeon | 11 | VM | M |
| Orthopaedic | Surgeon | 31 | RT | M |
| Obstetrics/gynae | Surgeon |  | RT | F |
| Plastics | Surgeon |  | RT | M |
| General surgery | Theatre personnel | 23 | VM | F |
| General surgery | Theatre personnel | 26 | VM | F |
| General surgery | Theatre personnel | 4 | VM | M |
| IPC | Nurse |  | RT | F |

**Table S2 Sample coding framework – Knowledge and skills theme**

| **Quotation** | **Code** | **Category** | **Theme** |
| --- | --- | --- | --- |
| **A&E nurse**  **Any policies or guidelines that you are aware that applies to surgical site infection prevention?**  No.  **Anaesthetist**  There seems to be not much standardisation between Trusts as well and sometimes it’d be nice to know why, it would be nice to know why one Trust prefers a Cephalosporin over a Penicillin for the exact same pathology in the exact same patient. From my own education and for the education of others that may be useful, and just communication really.  **Orthopaedic consultant**  That’s where it will tend to get brought up first. Now obviously if you’re missing from that meeting you may miss that but usually if there’s something important we will as colleagues inform each other.  **Tissue viability nurse**  There is the Trust policy for surgical site infections, which I'm aware of and it does state in it if they suspect a surgical site infection they're to refer to tissue viability… I'm not sure a lot people know about them to be honest, I don't know how well the surgical site infection guideline, how, whether people really know that it's there or follow by it, and then there's the NICE guidelines as well for a surgical site infection but I don't know whether it's very well known about.  **Vascular registrar**  we should have a scrape wound or take a fluid or tissue which, these kind of the guidelines were not, I wasn't aware of it, the wound swab.  No. So the audit of the antimicrobial prophylaxis, we got the feedback that we're giving the wrong doses of vancomycin in terms, but the guidelines changed, in terms of the dose … so it has changed really and it's very helpful, so this was the feedback from the pharmacy on the ward, that we should follow the guidelines which were changed recently, otherwise the prophylaxis is still pending and it's due to be changed 2019 so in the middle of this prophylaxis change. | Lack of information about policies, policy differences or changes | Awareness of policies | Knowledge and skills |
| **Anaesthetist**  I think it’s people who aren’t familiar with the antibiotic prophylaxis for the Trust could be more accessible, I know it’s on the source but if, if you had it actually written down for people without having to access a computer within the certain theatres where *especially these occur,* that could be of use.  Again I think ease of access to information, so for antibiotic policy it does need to be easier, it’s a big, big wordy document. I know that there’s, in other hospitals in the private sector, there’s a number of apps that you can use and it’s very easy, you literally just tap on it for that institution and Trust.  **Orthopaedic consultant**  I think there are some policies on the trust intranet. I have to say the trust intranet is absolutely full of policies which I don’t bother reading. I will do what we’re told to do if it is discussed in our audit meeting or, for example by the ward matron or whoever is the leader on it.  **Specialist nurse**  but anyone that nurses on the ward wouldn't have an idea of how to search up on the intranet for where the SSI protocol is. So I think it should be more easy at hand.  **Theatre personnel 1**  We have policies (inaudible) because we have a policy for everything that we do. And they all update online here so it’s accessible to all staff to be able to go in and check once you *go on* the source. You have more than enough, but it’s now the time for individuals to really go and search or ... | Policy information is difficult to access |
| **Critical care nurse**  So, if I do a wound care dressing as what I knew from university when your studying how to do it from washing your hands, to removing the dressing, to washing your hands again and preparing all your sterile field and what kind of, this is are all like the basic that I’ve learned from taking the degree  I don’t think this is standard across nurses, I don’t think so, because we don’t, I cannot remember that someone has shown me that this is how you should do wound care.  **Scrub nurse**  So, when I scrub we always have to scrub properly, I mean with the aseptic technique. So, as I’ve learned in uni | Relying on previous training, staff have different practices | Staff training, experience and confidence |
| **Vascular registrar**  what I notice also if the nurse doesn't want to do the dressing they will be very reluctant to call you when the dressing is down and they won't do it, they won't take it down, and at 6pm it's very difficult to get the dressing down because everyone is preparing for the handover, it's not fair on the staff as well or on the patient wait, God knows for how long, until midnight to have the dressing done, so either you do it yourself or it won't be done. | Lacking in confidence in their practices |
| **Critical care nurse**  when I do the dressing no one is there, so anyone who’s checking the dressing in that closed curtains, actually in my head I don’t know how they’re doing it. Do they do it the way I do, is it my practice it is the best, I don’t also know. | Unawareness of peer practices |
| **Scrub nurse**  I was telling you in my previous job we didn’t have to do it. So, it wasn’t, it’s like a health care assistant who has to do it, they are special training, trained to do that. So, they know how to do that, I don’t know and when I came to this country they said you have to clean the theatre at the end of the procedure and you have to be sure at the beginning of the procedure the theatre is clean. Of course, I will be sure that the theatre is clean at the end, at the beginning of the procedure, I will try to do my best, but I have no clue about that.  **Critical care nurse**  like changing the wound dressing, if you are a nurse then you should do it properly and I don’t know maybe it’s good showing you staff who doesn’t have, especially in the ward there are new staff nurses, this is their first job, that they need to be aware of how to look after the wounds, maybe they don’t know how to do wound dressing, the simple things, | Performing tasks they haven’t been trained for |
| **Anaesthetist**  Certainly through the microbiology team and the infection control team if a patient is needing an upgrade on their antibiotics or are on a long course of antibiotics, they’re highlighted to them as well. So all this information is then covered into the SSI prevention group.  **Cardiac registrar**  we have meetings between different departments to discuss that. The microbiologist tends to give the majority of the advice with some input from clinicians, so it’s mainly the microbiologist who decides what the antibiotic prophylaxis is.  **Critical care nurse**  We have doctors there all the time that you can easily get and then when you refer the surgeon, we are the priority to be seen.  but we have tissue viability as well, which is a very strong team we have,  but the surgeon is there to give us some instructions in what to do with this wound, or there might be, they have to obviously, it’s not resuture, they can’t resuture it so, using of a VAC pump would be beneficial for the patient.  No, it’s our intensive care doctors, but I am aware that every day in ITU they have the microbiology meeting, when the microbiology people come  **Orthopaedic registrar**  We have post operatively, if we are in doubt so far we have ANPs, advanced nurse practitioners, we have a special nurse that is responsible for orthoplastic cases and also we’ve started dressing clinic twice a week for any query wound problem, they will come, they will be targeted with *members of* staff.  **Tissue viability nurse**  I don't think it's, the referrals that I see, I don't get referred that many but that doesn't mean to say that it's not, they're not occurring or not being reported but I know there has been a slight increase in the number of SSIs occurring recently.  **Vascular registrar**  According to micro consultants, swabbing of the wound is not appropriate, we should have a scrape wound or take a fluid or tissue which, these kind of the guidelines were not, I wasn't aware of it, the wound swab.  If they don't know what kind of dressing to use and the junior doctor or whoever is on the ward doesn't know what to do it, they won't do it, so they don't, they will feel more supported and will learn from this as well.  but if they have this nurse and if she helps to reduce the SSI rate this is perfect, so she can be there, she can teach the people to do it … which is beneficial for everyone. | Strong connections with specialists | Other specialists as a resource |
| **Cardiac registrar**  We have (nurse) who will pick up surgical site infections, and, but we will not stop swabbing just because (nurse)’s going to pick up a surgical site infection, and I don’t think people will change their swabbing behaviour, especially because the nurse on the ward doesn’t care if (consultant) has a high rate of wound infection, they just want to treat the patient, and the way we’ve separated the person who’s most affected from the actual actions that need to be taken.  **Orthopaedic registra**  I think it is well organised team for surgical infection site in our trust starting from the nurses and to surgeon between, for example in our department of orthopaedic as well as the plastic surgeon and the microbiology department and as well as pharmacy.  **Anaesthetist**  that is the literature that’s printed and we’ve stuck with that and that has been questioned by senior people within microbiology that at a multidisciplinary meeting they decided to go with that evidence. | Advantages of multidisciplinary working |
| **Vascular registrar**  When I spoke to the matron about this, maybe it's worth trying to point out the nurse who'll be doing all the dressings just to reduce the wait, she told me that it would never work here because it will, not decondition, but it will deskill the other nurses  But it wasn't supported by the matron at the time and now, after a year, when the bombshell was blown, it's just everyone is, she just needs to use the golden part, have this wound dressing which everyone is very reluctant to have that person on the ward because it's not a part of their culture how it works here. | Access to specialists de-skills non-specialists |
